# Late-Onset Ornithine Carbamoyltransferase Deficiency Accompanying Acute Pancreatitis and Hyperammonemia

**DOI:** 10.1155/2013/903546

**Published:** 2013-08-29

**Authors:** Marcel Cerqueira Cesar Machado, Gilton Marques Fonseca, José Jukemura

**Affiliations:** ^1^Department of Clinical Emergencies, LIM 51, University of São Paulo School of Medicine, Rua Peixoto Gomide, 515 Conjunto 134, 01409-001 Bela Vista, SP, Brazil; ^2^Department of Gastroenterology, Digestive Surgery Division, University of São Paulo School of Medicine, Avenida Doutor Enéas de Carvalho Aguiar 255, Instituto Central, 9° Andar, Sala 9074, 05403-900 Cerqueira Cesar, SP, Brazil

## Abstract

Hyperammonemia related to urea cycle disorders is a rare cause of potentially fatal encephalopathy that is encountered in intensive care units (ICUs). Left undiagnosed, this condition may manifest irreversible neuronal damage. However, timely diagnosis and treatment initiation can be facilitated simply by increased awareness of the ICU staff. Here, we describe a patient with acute severe pancreatitis who developed hyperammonemia and encephalopathy without liver disease. Urea cycle disorder was suspected and hemodialysis was initiated. Following reduction of ammonia levels, subsequent treatment included protein restriction and administration of arginine and sodium benzoate. The patient was discharged to home after 47 days with plasma ammonia within normal range and without neurological symptoms. In clinical care settings, patients with neurological symptoms unexplained by the present illness should be assessed for serum ammonia levels to disclose any urea cycle disorders to initiate timely treatment and improve outcome.

## 1. Introduction

Hyperammonemia is a frequent complication of liver failure or portal-systemic shunt implantation. In patients without liver failure or portal-systemic shunt, hyperammonemia may result from urea cycle disorders, which can disrupt the hepatic disposal process that otherwise clears the toxic ammonia by-product from protein or amino acid breakdown. 

Clinical study has revealed that deficiency of any of the five primary urea cycle enzymes in infants results in ammonia accumulation that manifests as severe neurological symptoms culminating in coma and death. In adults, mild or partial deficiency of any of the urea cycle enzymes has been attributed to severe illness- or stress-induced increases in protein catabolism or protein overload [1]. OTC deficiency is the most common urea cycle disorder detected in adults, but this patient cohort presents with highly variable phenotypes [2]. Here, we describe a patient with infected necrotic acute pancreatitis that developed severe acute hyperammonemia with coma following surgical drainage who was effectively treated with sodium benzoate, arginine, and hemodialysis.

## 2. Case Report

**Figure 1:**
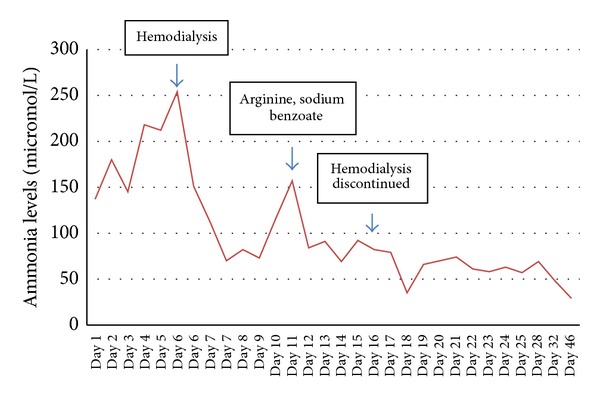
Effect of hemodialysis, arginine, and sodium benzoate on ammonia levels in late-onset OTC deficiency.

A 49-year-old male with biliary acute pancreatitis was admitted to another hospital's intensive care unit (ICU) for 13 days. After discharge, the patient presented to our hospital with high fever, and a large pancreatic abscess was detected by computed tomography (CT) scanning. Upon admission, the patient underwent necrosectomy, cholecystectomy, and abscess drainage in a single surgical setting. Seven days later, he required an additional surgical drainage. Parenteral nutrition was initiated, and on the 8th day the patient developed progressive lethargy, confusion, and coma requiring intubation. Laboratory tests indicated hyperammonemia (137 micromol/L) and normal liver function with enzyme levels and serum bilirubin within normal range. CT and magnetic resonance imaging (MRI) revealed no brain lesions. Lactulose treatment produced no improvement in clinical signs, and the patient remained in deep coma with ammonia elevated to 254 micromol/L. Since there was no evidence of liver disease, hyperammonemia caused by disruption of urea cycle due to an enzyme deficiency was suspected as the etiology of the patient's mental disruption. Since there was no time to wait the results of amino acid analysis, with brain damage risk, empiric treatment was started. Accordingly, parenteral and enteral nutrition were replaced with a protein-free diet. Continuous venous hemofiltration with dialysis was initiated. Thirty-six hours later, the ammonia level had decreased to 82 micromol/L and the patient's mental status had improved. The patient was weaned from the respirator and completely awake. However, hemofiltration discontinuation was accompanied by deterioration in mental status. Since OTC deficiency is the most common defect reported in late-onset hyperammonemia, sodium benzoate (3 g) and arginine (3 g) were administered every four hours via nasogastric tube. The mental status improved, and serum ammonia levels returned to normal. Plasma amino acid analysis showed reduced citrulline (13 micromol/L, normal: 16–51), severely reduced arginine (17 micromol/L, normal: 43–407), elevated glutamic acid (165 micromol/L, normal: 10–97), and elevated aspartic acid (8 micromol/L, normal: 1–4). Urinalysis showed elevated orotic acid (1.8 mmol/mol of creatinine, normal: 0.4–1.2). Late-onset OTC deficiency was diagnosed. The protein-free diet was gradually replaced with a protein diet, and no increase in ammonemia was detected. After 47 days, the patient was discharged to home with medication and counseling on diet. The final serum ammonia levels were within normal range (Figure 1). Four months after discharge, the patient re-presented with hyperammonemia and elevated liver enzymes. Arginine administration improved the serum ammonia levels.

## 3. Discussion

Under normal physiologic conditions, ammonia is converted to urea in the liver by five enzymes: carbamoyl phosphate synthase 1 (CPS1), ornithine transcarbamylase (OTC), argininosuccinic acid synthetase (ASS1), argininosuccinic acid lyase (ASL), and arginase (ARG) [3]. A cofactor-producing enzyme, N-acetyl glutamate synthase (NAGS), is also believed to contribute to the urea cycle mechanism according to the finding that a NAGS deficiency yields a phenotype identical to CPS1 deficiency; specifically, it was found that in NAGS absence, CPS1 is rendered inactive [4].

In newborns, hyperammonemia is usually due to severe deficiency or even absence of activity of any of the five enzymes of the urea cycle or the NAGS cofactor. Urea cycle disorder affects ~1 of 8200 live births in the United States [3]. Patients present with rapidly developed lethargy, hypothermia, hyperventilation, cerebral edema, seizures, and coma. However, these symptoms may not be recognized by treating physicians or may develop after hospital discharge. In adults, partial enzyme deficiency-related hyperammonemia may be triggered by an acute stress event or infection accompanied by a high-protein diet, as in our patient.

OTC catalyzes the synthesis of citrulline from carbamoyl phosphate and ornithine [3]. OTC deficiency produces a wide spectrum of genetic defects with different phenotypes; moreover, a variety of enzyme deficiency is found in patients with late-onset disease [2, 5]. In our patient, the hyperammonemia did not manifest any long-term complications, indicating that the patient may be heterozygote. His symptoms followed the precipitating factors of infected pancreatic necrosis, surgical debridement, and high-protein diet. The OTC deficiency diagnosis was based on decreased citrulline and arginine levels and increased glutamic and aspartic acids in plasma and increased urinary orotic acid [1, 3]. However, no increase in plasma ornithine levels was observed [6]. Definitive diagnosis can be obtained only by liver biopsy to measure OTC enzyme activity and mutation analysis [1]. In our patient, plasma aminoacid analysis and increased urinary orotic acid were consistent with the diagnosis of OTC deficiency. Liver function markers and the macroscopic intraoperative appearance of the liver were normal. 

In general, hyperammonemia treatment is based on the following objectives: decreasing waste products from endogenous protein breakdown by reducing the nitrogen intake; minimizing protein catabolism, and providing substrates lacking in the urea cycle and substances that may facilitate ammonia removal from the blood [3]. Arginine activates the urea cycle by activating NAGS [3] and should be administered as soon as the diagnosis is suspected. Activation of alternative pathways by sodium benzoate (as in our case), sodium phenylbutyrate, or glycerol phenylbutyrate can control the increase in serum ammonia [7, 8]. Liver transplantation is considered only in patients with recurrent hyperammonemia or resistant to medical conventional therapy. Decision for liver transplantation is also based on the extent of brain and liver damage (in argininosuccinate lyase deficiency). Levocarnitine is useful in hyperammonemia associated to primary carnitine defieiency usually associated with hypoketotic hypoglycemia, cardiomyopathy, hepatomegaly, and increased transaminases serum levels. Levocarnitine may also be useful in hyperammonemia due to administration of valproic acid [9, 10]. However, patients, like ours, with the acute form of the disease require hemodialysis or hemodiafiltration to quickly remove ammonia from the circulation and overcome the potentially lethal consequences. Though no empirical-based guidelines have been established for indications of hemodiafiltration or hemodialysis, patients with ammonia serum levels three-times above the upper limit or with encephalopathy are good candidates for these interventions. In our patient, however, hemodiafiltration was indicated at a late stage when serum ammonia level reached 254 micromol/L and coma had already occurred; even so, the patient had an uneventful recovery. 

Since ammonia is a gas, its rapid removal by hemodiafiltration is not associated with osmotic problems and no special care must be taken to avoid dialysis disequilibrium syndrome [11]. Long-term urea cycle medication, such as glycerol phenylbutyrate and sodium phenylbutyrate, is considered useful for clinical management of pediatric patients. However, after the acute episode in our adult patient, arginine and sodium benzoate were administered for only two weeks, at which time the normal diet was resumed. At follow-up two months after discharge, the serum ammonia level was within the normal range (29 micromol/L). At the four-month follow-up, however, laboratory tests indicated hyperammonemia and elevated liver enzymes. The cause of this disorder may be related to deficiency of downstream intermediates, such as arginine and/or arginine-derived intermediates [1]. In our patient, the diagnosis of late-onset urea cycle disorder was of paramount importance for appropriate treatment and successful recovery. However, even in late-onset hyperammonemia there are contraindications for certain drugs. For example, valproic acid (Depakene or Depakote) should be avoided due to its action on the urea cycle enzyme CPS1. Likewise, intravenous steroids, administration of large amounts of proteins or amino acids, excessive protein restriction, prolonged starvation, and of course conditions of excessive stress are potentially detrimental. In the case of unavoidable stress conditions, careful monitoring of serum ammonia is needed. In clinical care settings, patients with neurological symptoms unexplained by the present illness should be assessed for serum ammonia levels to disclose any urea cycle disorders that are amenable to treatment with favorable clinical outcome.
